# Effect of Different Cellulose Fillers on the Properties of Xanthan-Based Composites for Soil Conditioning Applications

**DOI:** 10.3390/ma16237285

**Published:** 2023-11-23

**Authors:** Alessandro Sorze, Francesco Valentini, Jasna Smolar, Janko Logar, Alessandro Pegoretti, Andrea Dorigato

**Affiliations:** 1 Department of Industrial Engineering, University of Trento and INSTM Research Unit, Via Sommarive 9, 38123 Trento, Italy; 2 Faculty of Civil and Geodetic Engineering, University of Ljubljana, Jamova cesta 2, 1000 Ljubljana, Slovenia

**Keywords:** Xanthan gum, cellulose, soil conditioner, water absorption, forestry, agriculture

## Abstract

The aim of this study was to investigate the effect of different types of natural cellulose-based fillers on the properties of Xanthan gum (XG) in order to develop novel bio-based soil conditioners (SCs) that could be used in forestry and agricultural applications. Rheological measurements highlighted that SCs with cellulose fillers characterized by a high aspect ratio and low oxide ash content exhibited an average increase of 21% in yield stress compared to neat Xanthan gum. The presence of cellulose fillers in the composites resulted in a slower water release than that of neat XG, limiting the volumetric shrinkage during the drying process. Furthermore, an analysis of the water absorption and water retention capacity of soils treated with the different SCs was carried out, demonstrating that the addition of 1.8 wt.% of SC with optimized composition to the soil led to an increase in water absorption capacity from 34% up to 69%. From the soil water retention curves, it was observed that the addition of SCs significantly increased the amount of water effectively available for plants in the area between field capacity and permanent wilting point (100–1000 kPa). From practical experiments on grass growth, it was observed that these SCs improved the water regulation of the soil, thus increasing the probability of plant survival under drought conditions.

## 1. Introduction

In recent years, due to climate change, an increase has been observed in the recurrence (i.e., reduction of recurring time) of extreme events such as floods and droughts with serious consequences for humans and nature [1,2,3]. It is estimated that in Italy, the extreme drought of 2022 caused around 6 billion € of damages, while in 2023, the flood in Emilia Romagna caused over 9 billion € of damages [4]. The European Environmental Agency estimated that from 2008 to 2017, the surface area in Europe with a very high sensitivity to desertification increased from 234 to 411 thousand km^2^ [5]. Desertification is the result of an increase in frequency, intensity, and duration of drought periods that can overcome the recovery ability of vegetation, leading to soil degradation with further exacerbation of negative phenomena such as sand and dust aerosols, greenhouse gas fluxes, an increase in surface temperature, and soil sealing [6,7,8].

Several strategies can be applied to mitigate the effects of heat and drought on soil: revegetation/afforestation, plantation of street trees, establishment of plant covers, green roofs, use of porous pavements in urban areas instead of asphalt or concrete, adoption of rainwater reservoirs, and rainwater harvesting techniques [9,10,11,12,13,14,15,16]. Regarding revegetation and afforestation, there is a need for technological solutions able to support planting and forestry operations, hence promoting the survival rate of growing plants [17,18,19].

Among the different solutions to these problems, the use of soil conditioners (SC) is very promising in terms of both performance and cost. Soil conditioners are products that can be mixed with the soil in the planting pit to enhance its physical, chemical, mechanical, and water-regulating characteristics [20,21]. Commonly, in agriculture, the most widely used soil conditioners are products based on compost or manure that act as fertilizers to provide organic substances and nutrients to plants [22,23,24]. To counteract the problems caused by drought and water shortage, one of the main requirements for these products is the ability to absorb large amounts of water; therefore, superabsorbent hydrogels are widely used for this type of application [25,26,27,28,29,30,31,32,33,34]. Several studies have demonstrated that soil conditioners can effectively reduce water consumption during irrigation, lower plant mortality rates, and consequently promote plant growth [35,36,37]. However, traditional soil conditioners often rely on synthetic polymers, primarily polyacrylamide, which come with significant drawbacks. In fact, these synthetic materials have limited bio-degradability and release harmful by-products into the soil, posing risks to both the environment and human health [25,38,39,40]. To address these concerns, researchers have considered various biopolymers as potential alternatives to traditional plastics for SCs [41,42,43,44,45,46,47].

Biopolymers are polymeric materials derived from renewable sources and/or biodegradable [48,49,50,51,52]. They encompass a variety of polymers, such as polysaccharides (e.g., cellulose) and proteins (e.g., gelatin, casein, and silk). Additionally, they can be chemically synthesized from bio-derived monomers (e.g., polylactic acid [53]) or through microbial activity (e.g., Xanthan gum, polyhydroxyalkanoates). Biopolymers are recognized for their eco-friendly characteristics and have found widespread use both in food and medical applications [54,55]. Among them, Xanthan gum stands out as particularly promising for soil conditioning purposes due to its biodegradability, filmability, soil strengthening capability, and excellent water-absorbing properties [56,57,58,59,60]. Xanthan gum is a polysaccharide produced by *Xanthomonas campestris* through aerobic fermentation [61]. It consists of two glucose units, two mannose units, and one glucuronic acid unit, which primarily form helical structures [62,63]. To further enhance the performance of biopolymers, fillers based on wood or cellulose fibers can be incorporated. These fillers offer the potential to optimize SC characteristics in terms of water absorption, water retention, and mechanical stability [64,65,66,67]. As demonstrated by Sorze et al. [68], the combination of Xanthan gum with cellulose fibers resulted in increased soil water regulation properties. However, no other studies on similar materials applied for soil conditioning applications can be found in the literature. Therefore, the aim of this work is to further study the effect of different types of natural cellulose-based fillers on the properties of Xanthan gum in order to develop novel multifunctional bio-based composites to be used as soil conditioners (SCs) for agricultural and forestry applications.

## 2. Materials and Methods

### 2.1. Materials

The Xanthan gum, commercial grade with purity > 91% and molecular weight (Mw) ∼1.0 × 10^6^ g/mol, was provided in the form of fine powder by Galeno Srl (Prato, Italy) and used as received. Different cellulose fillers were kindly provided by J. Rettenmaier and Söhne Gmbh (Rosenberg, Germany). Their main characteristics are listed in Table 1. Commercial wood fibers, Steico Flex 036, were kindly provided by Technische Hochschule Rosenheim (Rosenheim, Germany) and milled for 30 s before use.

Two types of soils were used in this investigation:

- (S1) A top soil layer sampled from a garden in the Department of Industrial Engineering of the University of Trento (46.06° N, 11.15° E, altitude 398 asl) and characterized by Fondazione Edmund Mach (San Michele all’Adige, Trento, Italy). The main properties of this soil are listed in Table 2.
- (S2) A top soil layer sampled in Alpine forests from the Ljubelj area (Slovenia, E(D96/TM) 443431, N (D96/TM) 144159, altitude 1100 asl) [69]. This is fine-grained soil and can be classified according to USCS (ASTM D2487-17 [70]) as organic silt of high plasticity (OH). The average value of natural gravimetric water content, determined according to ISO 17892-1 [71], is 42.9%. The main properties of this soil are listed in Table 2.

In addition, two different commercial soil conditioners based on potassium polyacrylate were also used as benchmarks. Idrogea was purchased by Endofruit Srl (Verona, Italy) and Be-Grow Boost M, kindly provided by the University of Freiburg (Germany) and produced by Be-Grow GmbH (Neustadt an der Weinstraße, Germany). According to the technical datasheet, the concentrations of these products to be used in the dry soil are 0.13 wt.% and 0.4 wt.%, respectively.

**Table 1:** Main features of the cellulose-based fillers utilized in this work [72,73].

Type of Filler	Basic Raw Material	Cellulose Content (%)	Oxide Ash (850 °C, 4 h) (%)	Average Fiber Length (µm)	Aspect Ratio
Arbocel R	Pure cellulose	>99	0.5	200–300	9.9
Arbocel FT 400	Technical cellulose	75	2	1200	34.4
Arbocel ZZC 500	Technical raw cellulose	80	15	400	8.8
Cellugrün	Technical raw cellulose	80	15	1400	31.1
Arbocel ZZ 8-2 CA1	Technical raw cellulose	50	50	1000	22.2
Arbocel Adsorb 2	Cellulose dextrose derivate	-	-	30–250 (particles)	- *
STEICO flex 036 (milled)	Wood fibers	-	-	9000–30,000	25

* Arbocel Adsorb 2 is constituted by very fine powder, with no info on the aspect ratio provided by the supplier.

**Table 2:** Results of the chemical analysis of the soil S1 and soil S2.

Determination	Soil (S1)	Soil (S2)
Sand (2.0–0.05 mm)	412 g/kg	202 g/kg
Silt (0.05–0.002 mm)	458 g/kg	493 g/kg
Clay (<0.002 mm)	130 g/kg	305 g/kg
pH (in water ratio 1:2.5)	8.1	7.5
Total limestone	349 g/kg CaCO_3_	17 g/kg CaCO_3_
Active limestone	15 g/kg CaCO_3_	-
Organic substance	33 g/kg	82 g/kg
Assimilable phosphorus	27 mg/kg P_2_O_5_	<60 mg/kg P_2_O_5_
Potassium	166 mg/kg K_2_O	<100 mg/kg K_2_O
Magnesium	317 mg/kg MgO	-

### 2.2. Soil Conditioners Preparation

Soil conditioners were produced by dissolving Xanthan gum powder in hot water (T = 60 °C) at a concentration of 4 wt.%. The mixing was carried out using a Dispermat^®^ F1 mixer (VMA-Getzmann Gmbh, Reichshof, Germany), operating at 5000 rpm for 15 min, in order to obtain a homogeneous mixture without lumps. The fillers were gradually added during the mixing operations at a concentration of 2 wt.% (relative to the water solution), according to the previous work of Sorze et al. [68]. In this way, it was possible to obtain seven different compositions, as reported in Table 3. The neat XG was also studied as a reference. For some analysis, samples were used in the dried state, and therefore, the produced composites were put in an oven at 50 °C for 72 h. Finally, the prepared materials were ground for 3 min using a Piovan^®^ RN166/1 granulator (Piovan SpA, Venice, Italy) to obtain samples in the form of dried powder. 

### 2.3. Experimental Techniques

#### 2.3.1. Rheological Properties

The rheological tests were performed in order to measure the viscosity and the shear stress in a shear rate interval between 0.1 and 100 s^−1^ of the prepared samples in the wet state. A Discovery Hybrid Rheometer DHR-2 (TA Instrument, New Castle, DE, USA), operating in plate-plate configuration with a plate gap of 1 mm and at a constant temperature of 30 °C, was used. From these tests, it was possible to determine the yield stress *σ_y_* of the wet samples by fitting the rheological curves with the Casson model [74], described by Equation (1):

(1) $$\sigma^{1/2}=\sigma_{y}^{1/2}+(\eta {\dot{\gamma })}^{1/2}$$

where *σ* is the shear stress, *η* is the shear viscosity, and $\dot{\gamma }$ is the shear rate. The value of the yield stress was detected as the intercept with the *y*-axis of the linear fit of the *σ* data in the strain rate range from 0.3 to 2 s^−1^.

#### 2.3.2. Fourier-Transformed Infrared Spectroscopy (FT-IR)

Fourier-transformed infrared spectroscopy (FT-IR) tests were conducted in attenuated total reflectance (ATR) mode using a PerkinElmer Spectrum One spectrometer (PerkinElmer, Waltham, MA, USA), in the wavelength number range of 650–4000 cm^−1^, obtaining each spectrum from the superposition of four scans. Neat Xanthan gum (XG) and the different fillers were tested.

#### 2.3.3. Light Microscopy

To study the morphology of the prepared soil conditioners in their dried state, light microscopy observations were performed using a Nikon SMZ25 light microscope (Nikon, Tokyo, Japan), equipped with a Nikon DS-Fi2 digital camera (Nikon, Tokyo, Japan). 

#### 2.3.4. Moisture Absorption and Water Retention Capability

Moisture absorption tests were carried out on the prepared samples, which were conditioned for 48 h in the oven at 50 °C. The samples, stored in a sealed box at RH = 80% and T = 25 °C, were periodically weighed. The mass was measured with a Gibertini E42 balance (resolution of 0.1 mg). The moisture absorption (*MA%*) was calculated according to Equation (2):

(2) $$MA\%=\frac{m_{wet}-m_{dry}}{m_{dry}}\cdot 100$$

where *m_wet_* is the mass of the sample in the humid state, and *m_dry_* is the mass of the dried material. For the evaluation of water retention, 16.5 mL of demineralized water was added and mixed with 1 g of the dried samples. Then, wet samples were placed under constant thermo-hygrometric conditions (T = 25 °C, RH = 50%). In this way, by periodically measuring the weight of the samples, it was possible to calculate the residual water content (*RW%*), i.e., the water retention capability, according to Equation (3):

(3) $$RW\%=\frac{m_{i}-m_{dry}}{m_{wet}-m_{dry}}\cdot 100$$

where *m_i_* is the mass of the sample during drying.

#### 2.3.5. Application on Soil

In this paper, not only the characterization of XG-based SCs is performed but also the characterization of soils mixed with SC in order to study the water holding capacity, water retention, and soil water retention capacity. Part of the research program was thus conducted in laboratory 1 (Trento) and part in geotechnical laboratory 2 (Ljubljana). For this reason, soil-SC mixtures were tested by different but comparable and complementary methods on two different soils.

##### Evaluation of the Water Holding Capacity (WHC) of Soil S1

The evaluation of the water holding capacity of the soil S1 treated with dry SCs was carried out by adapting the procedure of Yu et al. [75]. The test was performed on soil sieved through a 1.8 mm sieve and with an initial measured water content of 10 wt.%. The sieved soil samples (20 g) were mixed with 1.8 wt.% of the different SCs. The untreated soil was tested as a reference, and the soil mixed with commercial products Idrogea and Be-Grow Boost (according to the conditions specified in Section 2.1) was also analyzed as a comparison. Moreover, to demonstrate the positive effect of adding fillers to Xanthan-based solutions, a soil mixed with 1.2 wt.% of neat Xanthan gum powder (which is the amount of Xanthan gum present in the SC samples) was also tested. All the samples were enclosed in perforated polyethylene films (16 cm × 16 cm × 0.1 mm), permeable to water but not to the soil. The soil envelopes were weighed (*m_dry_*) and then placed in demineralized water for 15 min. Then, excess gravitational water was drained, sample weights were recorded (*m_wet_*), and the WHC was calculated using Equation (2).

##### Evaluation of the Water Retention Capacity of the Soil S1

The influence of SCs added to the soil on the water retention capacity was evaluated according to the procedure proposed by Ni et al. [76]. 50 g of dried soil was sieved through a 1.8 mm sieve and mixed with the 1.8 wt.% of the dried SCs. 50 mL of demineralized water was then slowly added, and the resulting samples were weighed. Untreated soil, soil treated with commercial products, and soil mixed with neat Xanthan gum powder were also tested as a reference. The samples were then conditioned at 30 °C in an oven and periodically weighed. The residual water content, which corresponds to the water retention capacity, was calculated using Equation (3).

##### Evaluation of the Water Absorption (wA) of Soil S2

The water absorption tendency of untreated and treated soil S2 was determined in accordance with DIN 18132 [77] (long-term test > 1 h). Due to the presence of organic content in the soil, samples were dried in the oven at 45 °C until a constant mass was reached. The samples were then placed in a desiccator and let cool to ambient temperature. Larger soil lumps were crushed to achieve soil samples with particles smaller than 0.4 mm. Tests were conducted on untreated and treated soil specimens with a dry mass of 1 g. Treated soil specimens were prepared from dry soil samples (d_max_ < 0.4 mm) mixed with dry soil conditioners SC_R, SC_CG, and SC_ZZC. The dosage of SCs was defined as the percentage of their mass to the dry mass of the soil: 0.4 wt.% (low dosage) and 1.7 wt.% (high dosage). To create reproducible conical specimens, the dry specimens were placed on a filter disc using the funnel. The measurements of time and volume of absorbed water started when the first particles dropped onto the filter disc. Water absorption was calculated using Equation (2) for a test duration of 24 h (w_A 24h_) and after the test was finished (specimen was fully wetted, and no further absorption of water was registered after two successive time intervals (w_A max_)). Although the specimen holder was covered with a glass stopper during the test, the measured values were corrected to account for evaporation. The evaporation rate was frequently determined by measuring the time changes in water volume in the measuring capillary without a specimen on the filter disc.

##### Determination of the Soil Water Retention Curve (SWRC) of Soil S2

**Figure 1:**
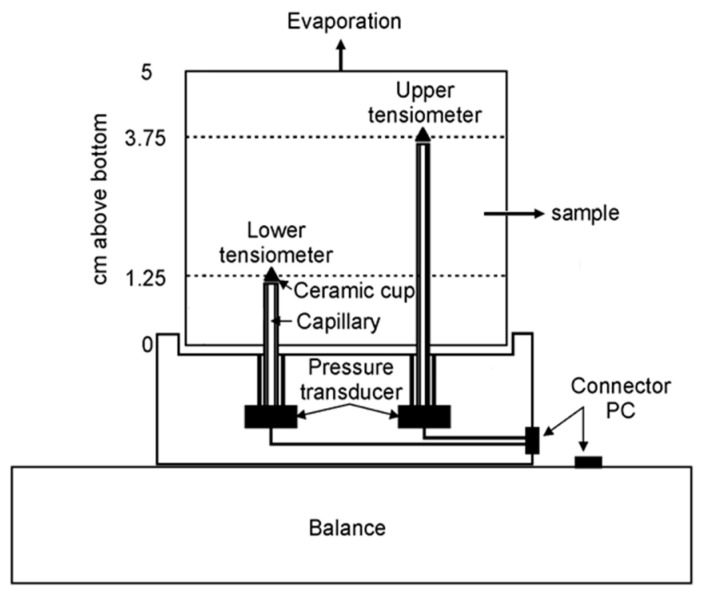
Schematic cross-section of the HYPROP evaporation method device (reprinted with permission from [80].

Soil water retention curves (SWRCs) were determined using the Hyprop evaporation method device (matric suctions up to approximately 100 kPa) and Dew Point Potentiometer WP4-T (total suctions higher than approximately 100 kPa). Figure 1 shows a schematic of the Hyprop device involving pressure head measurements at two depths (1.25 cm and 3.75 cm) within a 5 cm high saturated specimen with a diameter of 8 cm [78]. The evaporation rate was obtained by automated weighing. The assumption that changes in weight are equal to changes in the mass of water was used. This means that possible biodegradation during the investigation, which lasted at least 1 month, was not taken into account. Water retention values were estimated from the average gravimetric water content and the average pressure measured by the two tensiometers. Specimens were prepared from untreated and treated soil (mixtures). Homogeneous mixtures with SC_R, SC_CG, and SC_ZZC were prepared from the soil at natural water content with the addition of dry SCs and slightly compacted into the Hyprop mold. The dosage of SCs was equal to the dosage in samples for the determination of water absorption. The specimens in the mold were then saturated by immersing them in water to their 4/5 height. Volume changes were reduced by porous discs on both ends of the specimen (at the upper and lower surface), and a weight of 10 kPa was applied on the top of the specimen during the saturation process. After saturation was finished, tensiometers mounted on the Hyprop device were pushed into the specimen, and measurements started. To provide quasi-steady state conditions (constant flux and hydraulic gradient over the time interval), evaporation was slowed down by covering the specimens with a perforated plastic bag. In the high suction range, SWRC was measured using the potentiometer WP4-T, following the procedure described in ASTM D6836 [79]. The measurements were conducted on the remaining portion of the sample (soil or mixture), prepared for the specimens investigated in the Hyprop device. Using drippers, the samples were wetted to water content at or slightly below full saturation. At this point, the first specimen was prepared, and total suction was measured. The sample was then slowly dried stepwise at room temperature, and specimens were taken at various drying intervals. Before conducting the measurements, the prepared specimens were tightly sealed in special plastic containers for at least 24 h to ensure that the water content and the suction were equilibrated. Before the regular measurements started, the potentiometer WP4-T had been calibrated each day by using a standard solution of 0.5 M KCl. The dry mass of all specimens was determined after suction measurements by drying in an oven at 45 °C. 

##### Case Study Application

###### Evaluation of the grass germination in soil S1

**Figure 2:**
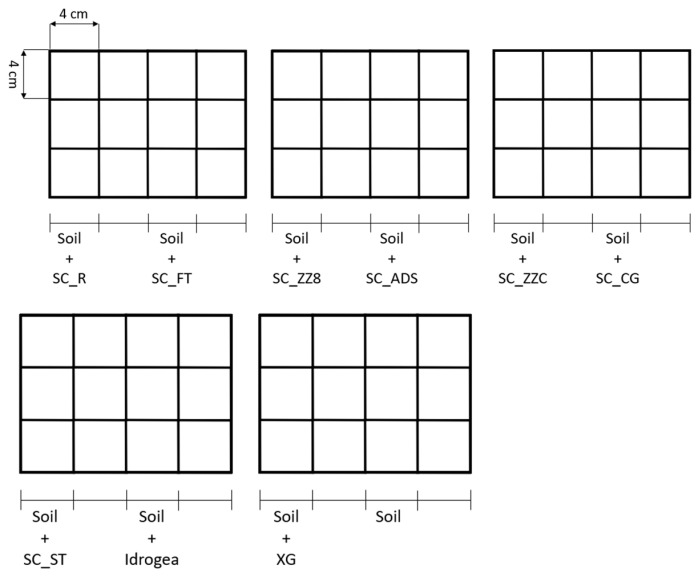
Schematization of the experiment for the evaluation of the grass growth with soil conditioners.

The produced Xanthan gum-based SCs were applied to the soil to investigate the effects of the biopolymers on plant growth. The test was performed on soil treated with the seven soil conditioners listed in Table 3 on the untreated soil S1 taken as reference, on the soil mixed with neat Xanthan gum powder, and on the soil mixed with the commercial product Idrogea. The mixing conditions were the same as described in Section Evaluation of the Water Holding Capacity (WHC) of Soil S1, and the experimental procedure was adapted from the work of Chang et al. [37]. Three pots (length 4 cm × width 4 cm × depth 5 cm) for each sample were used (a total of 30 pots), as shown in the schematization reported in Figure 2. In each pot, 15 grass seeds of *Nepeta cataria* (HortuSì Srl, Longiano, Italy) were planted. The seeds were covered with a thin layer of untreated soil (around 5 mm) and conditioned at RH = 30% and 25 °C. Each pot was watered daily with 2 mL of water, and no nutrients were applied. After 18 days, the seed germination, expressed as the ratio of germinated seeds over the planted seed, was calculated. 

## 3. Results and Discussion

### 3.1. Rheological Properties

**Figure 3:**
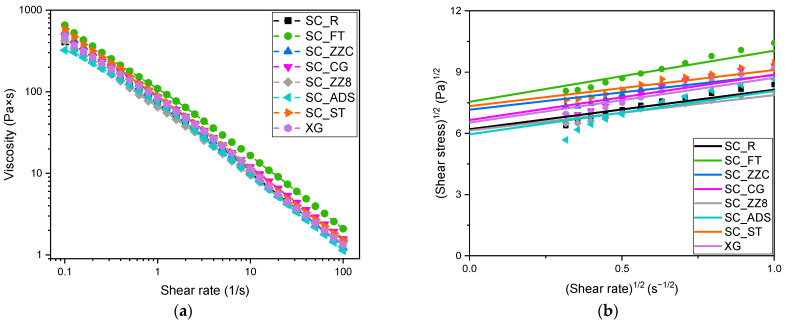
Rheological tests on the prepared SCs in the wet state. (**a**) Trend of shear viscosity with respect to the shear rate. (**b**) Linear interpolation of shear stress values for evaluation of the yield stress, using the Casson model (see Equation (1)).

Figure 3a shows the results of the rheological investigations performed on the prepared SCs in the wet state. Figure 3b and Table 4 display the yield stress values obtained by fitting the shear stress data using the Casson model (see Equation (1)).

From Figure 3a, it is possible to observe that rheological properties are closely related to the morphology of the fillers constituting the samples. For instance, fillers with a high aspect ratio (see Table 1) may interlace and form tangled networks [81]. Indeed, the addition of fillers with a high aspect ratio leads to an increase in both the viscosity and the shear stress with respect to the neat Xanthan gum (XG). In particular, SC_FT, which is constituted by the filler with the highest aspect ratio, is characterized by the highest viscosity. However, several factors besides aspect ratio are hypothesized to affect the viscosity of these samples; for example, the presence of oxide ash in the fillers may lead to a reduction of the viscosity and the shear stress. In fact, despite the good aspect ratio, sample SC_ZZ8 has the lowest rheological properties, which can be explained by considering the elevated content of oxide ash (50%) in this filler. Moreover, the sample SC_ADS is composed of very fine particles of cellulose dextrose derivate (30–250 µm) that may not form a good network with the Xanthan gum, resulting in poor rheological properties. From Figure 3b and Table 4, it is possible to notice that the values of yield stress are also influenced by the aspect ratio of the fillers and by the oxide ash content. In general, samples with a filler characterized by a high aspect ratio and low oxide ash content, like SC_FT, SC_ST, SC_CG, and SC_ZZC, exhibit higher yield stress with respect to neat Xanthan gum, evidencing an improvement of the gel network strength.

### 3.2. FT-IR Spectroscopy

**Figure 4:**
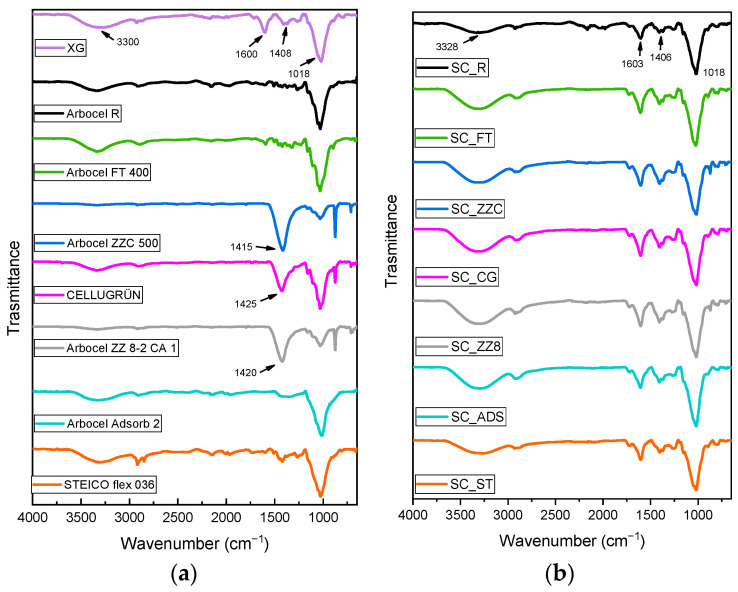
FT-IR spectra of (**a**) Xanthan gum and cellulose-based fillers and (**b**) the corresponding SCs.

In Figure 4a,b, the results for the FT-IR analysis performed on the Xanthan gum, fillers, and corresponding soil conditioners are displayed.

From Figure 4a, it is possible to compare the spectra of cellulose fillers with that of Xanthan gum. In particular, the broad absorption peak observed in the XG spectrum at around 3300 cm^−1^ corresponds to the O-H stretching vibration of the hydroxyl group, while the peaks at 1600 and 1408 cm^−1^ are associated with the asymmetrical and symmetrical C=O stretching vibration of carboxylate anion (-COO-), respectively. For Arbocel ZZC 500, Cellugrün, and Arbocel ZZ 8-2 CA 1, the absorption peak around 1420 cm^−1^ is more evident with respect to the other fillers. This can be explained by the very high oxide ash content of these fillers (see Table 1), associated with the presence of more C=O bonds. The characteristic peak at 1014 cm^−1^ for C-O stretching of primary alcohols is also observed for all the samples. From Figure 4b, it is clearly visible that the characteristic spectra of SC samples are very similar to each other and to that of neat Xanthan gum (XG), which is the main constituent of the soil conditioners. Moreover, the similar chemical structure of the cellulose fillers to the Xanthan gum does not allow for the identification of the significant differences between SC spectra.

### 3.3. Light Microscopy

**Figure 5:**
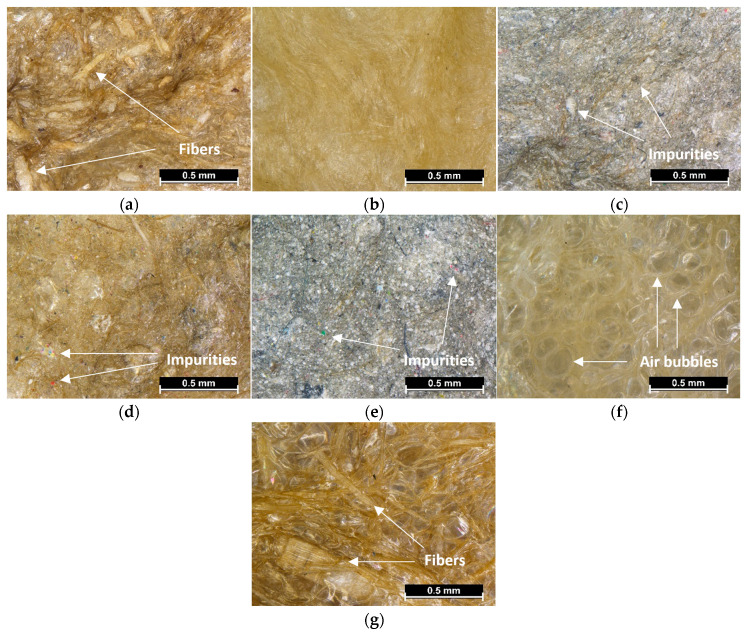
Light microscope images of the dried SCs: (**a**) SC_R, (**b**) SC_FT, (**c**) SC_ZZC, (**d**) SC_CG, (**e**) SC_ZZ8, (**f**) SC_ADS, and (**g**) SC_ST.

In Figure 5a–g, the light microscopy images for each dried SC are shown.

From Figure 5a,g, it can be observed that SC_R and SC_ST samples are composed of fillers with a wide distribution of fiber length, as also reported in Table 2. Figure 5f shows that the SC_ADS sample is full of air bubbles formed during the mixing process, and this may affect the final density of the material. From Figure 5c–e, it can be seen that SC_ZZC, SC_CG, and SC_ZZ8 show a lot of impurities that were already present in their cellulose fibers and, together with the high oxide ash content, contribute to the darker color of the image. Overall, it is possible to notice that for every sample, the structure appears quite homogeneous, without the presence of biopolymer lumps or filler aggregates. This indicates that a good mixing procedure was carried out, as also demonstrated by the good dispersion of the polymer and the fillers in the water solution. This homogeneous mixture leads to the formation of a network between the cellulose fillers and Xanthan gum structure that may result in a reinforcing and stabilizing effect of the composite when applied to the soil [82,83].

### 3.4. Moisture Absorption and Water Retention Capability

**Figure 6:**
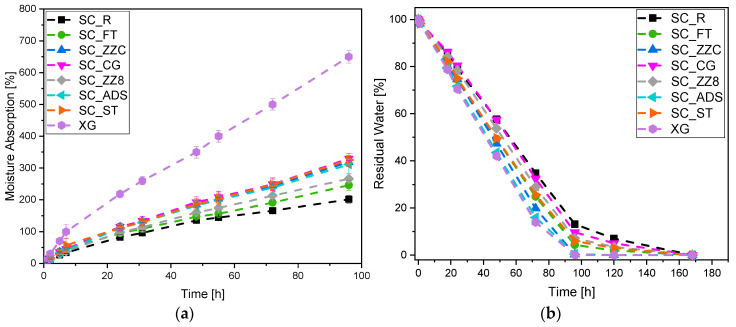
(**a**) Evaluation of the moisture absorption and (**b**) the residual water content for the different SCs.

In Figure 6a,b, the results of the moisture absorption and water release tests for the different SCs are displayed.

Figure 6a shows that the moisture absorption of the tested samples increases parabolically with time. Overall, the addition of cellulose fillers to the Xanthan gum solution leads to a decrease in the moisture absorption capacity. The observed behavior can be attributed to the chemical similarity between Xanthan gum and cellulose, which facilitates strong interfacial adhesion between the two components. This physical interaction can somehow hinder the diffusion of water molecules in the SC [65]. The test was conducted under high humidity conditions (Section 2.3.4); therefore, the analysis was stopped before it reached the plateau due to the formation of molds in the samples. Figure 6b displays that during the water retention test, there is an initial linear decrease in mass over time. In this case, the presence of the fillers in the samples leads to a slower water release compared to XG, which reaches complete drying after 96 h. For the other samples, this occurs between 120 h and 168 h (5–6 days). Furthermore, the presence of fillers in the solution helps to limit the volumetric shrinkage during the drying, which is also very significant for the XG sample. These tests indicate that the most promising compositions for a practical application are those that couple good moisture absorption capacity with a slow water release, such as SC_CG, SC_ST, and SC_ZZ8.

### 3.5. Application on Soil

#### 3.5.1. Evaluation of the Water Holding Capacity (WHC) of the Soil S1

**Figure 7:**
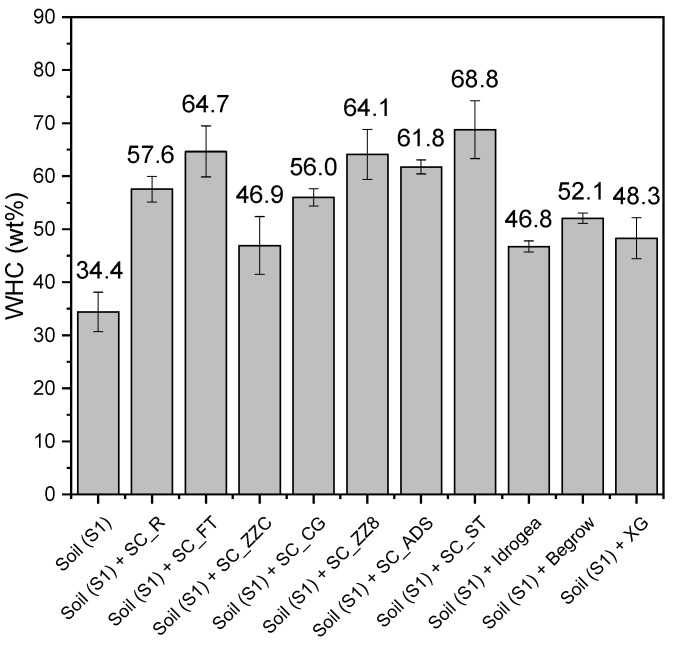
Water holding capacity of soil S1 with the different soil conditioners and commercial products.

The results of WHC testing on soil S1 mixtures with the various SC compositions are shown in Figure 7.

Figure 7 shows that the water holding capacity (WHC) of the soil increases when Xanthan-based soil conditioners are added. In particular, the reference untreated soil S1 has a WHC of 34.4%, which means that saturation is reached when 35 mL of water is poured into 100 g of dry soil. The addition of 1.8 wt.% of the prepared soil conditioners to the soil increases WHC values up to 68.8% (SC_ST sample), i.e., double that of the untreated soil. Moreover, Xanthan-based soil conditioners exhibit better water absorption properties than commercial products. In particular, the SC_ZZC sample has a water holding capacity as high as 46.9%, which is the lowest value among the cellulose-filled SCs. However, it is still comparable with that of soil treated with commercial products (Idrogea and BeGrow), which show WHC values of 46.8% and 52.1%, respectively. Moreover, the WHC value cannot be increased by further increasing the amount in weight of commercial products in the soil, as it was experienced that this would lead to a significant increase in soil volume with the formation of an incoherent gelatinous mixture of polymer and soil. In a practical application, this may lead to an evident swelling, which will be investigated in future work as part of geotechnical investigations of forest soils treated with developed SCs. Furthermore, for all the samples, the WHC values increase or are equivalent compared to neat Xanthan gum (48.3%), demonstrating the advantages of adding fillers to the biopolymer solution.

#### 3.5.2. Evaluation of the Water Retention Capacity of the Soil S1

**Figure 8:**
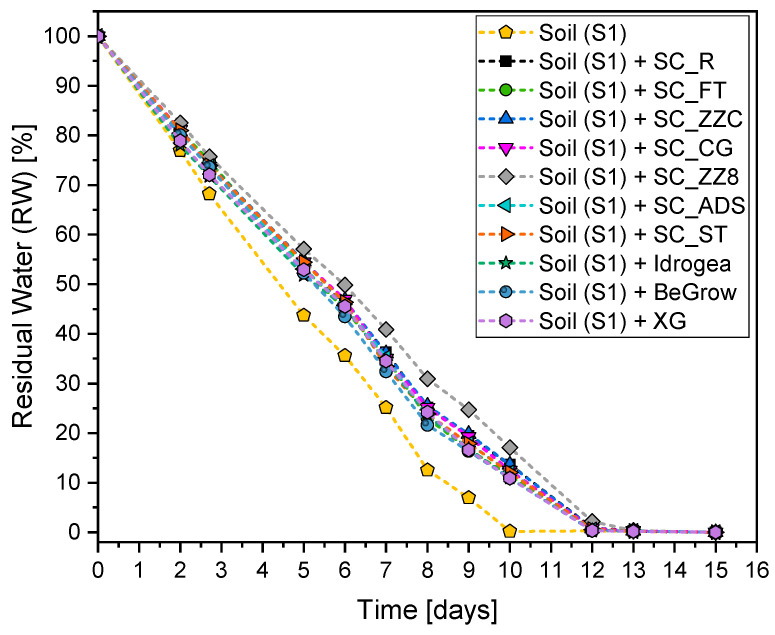
Water retention behavior of soil (S1) mixed with cellulose-filled soil conditioners and commercial products.

In Figure 8, the results of the evaluation of the water retention capacity of the untreated and treated soil are reported.

Figure 8 shows that the presence of soil conditioners leads to an increase in the water retention capacity compared to the untreated soil, which means a slowdown in the water evaporation rate from the soil. In particular, the untreated soil has a weight loss of 56.3% after 5 days and 93.2% after 9 days. For all the other samples, weight loss values are around 45.5% after 5 days and 80.5% after 9 days. SC_ZZ8 shows the best performance, with weight loss values of 48.1% and 80.8% after 5 and 9 days, respectively. Both commercial products lead to weight loss values of 47.7% and 83.6% after 5 and 9 days, respectively, higher than the average of the cellulose-filled compositions. This means that the rate of water evaporation of the cellulose-filled SCs is lower than that shown by commercial products. This means that composites based on Xanthan gum and cellulose fillers are more suitable to retain water for a longer time, and this is extremely important for plants in dry climate conditions. Practically, the delay to the dry state between untreated and treated soil is about 2 days.

#### 3.5.3. Evaluation of the Water Absorption (wA) of Soil S2

Table 5 presents the results of water absorption tests carried out on untreated and treated soil S2. The addition of SC generally increases the water absorption of soil. The increase depends on the amount and the type of SC. Activation of SC in soil mixtures does not happen immediately, which is reflected in the absorption time needed to achieve w_A max_. In particular, it is possible to observe that treated soil is able to absorb 1.5 times more water than untreated soil, with the highest values reached after 24 h using a high dosage of SC_R. It is also interesting to notice that the use of low amounts of SC_R does not provide any improvement with respect to untreated soil.

**Figure 9:**
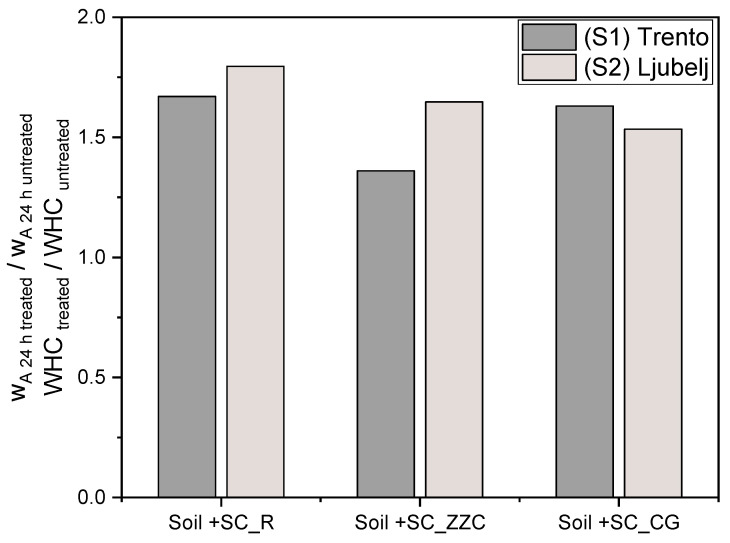
The influence of SCs on WHC and w_A 24h_ values of S1 and S2 soils.

These results are in good agreement with the data obtained for soil S1. Even though the investigations were carried out in two different laboratories, by adopting different experimental procedures and different soils, the influence of SCs on water holding capacity and water absorption was similar. This is confirmed by Figure 9, in which the relative improvement of w_A 24h_ and WHC values upon SC insertion for both S1 and S2 soils is reported.

#### 3.5.4. Determination of the Soil Water Retention Curve (SWRC) of Soil S2

**Figure 10:**
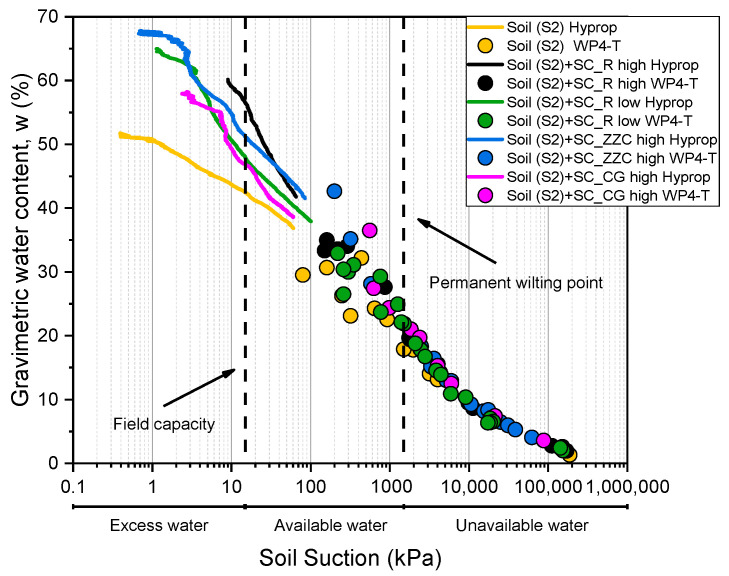
Soil water retention curves of untreated and treated soil S2.

Figure 10 shows the soil water retention curves of untreated and treated S2 soil. Results indicate that SC does not affect suctions higher than 2000 kPa, while there is an increase in available water in the range of low suctions up to 100 kPa when SCs are added to the soil. In the range of suctions between 100 kPa and 2000 kPa, the difference between suction of untreated and treated soil is low, within 5%, and this could be also a result of specimens’ inhomogeneity and preparation. For the assessment of available water for plant growth, two points of the SWRC are particularly important and are indicated in Figure 10 by black vertical dashed lines: field capacity at suctions between 10 and 33 kPa and permanent wilting point at suction approximatively 1500 kPa [84]. As can be seen from Figure 10, the addition of SCs has a beneficial effect on the available water for plants in the zone between field capacity and permanent wilting point. In the zone of excess water, the SCs increase the water content. During the saturation of treated soil specimens, swelling was observed. As a result of swelling, porosity increases, and consecutively, the water content of the saturated specimen increases. The initial density of the saturated specimen thus affects the position of SWRC. In Figure 10, the increase in energy needed to suck water from the treated soil can be observed in the range of low suctions (up to 100 kPa). At the same water content, the SWRCs of treated soil are shifted to the right compared to the SWRC of untreated soil. However, this does not represent a limitation for the use of SCs, because the phenomenon is observed in the area of excess water and at the transition to available water for plants, where sucking energy is low. Conversely, at high suction (>1000 kPa), corresponding to the condition of almost no available water for the plant (drought), the presence of SC in the soil does not increase the energy necessary to extract water from the soil with respect to the untreated soil.

#### 3.5.5. Case Study Application

##### Evaluation of the Grass Germination in Soil S1

**Figure 11:**
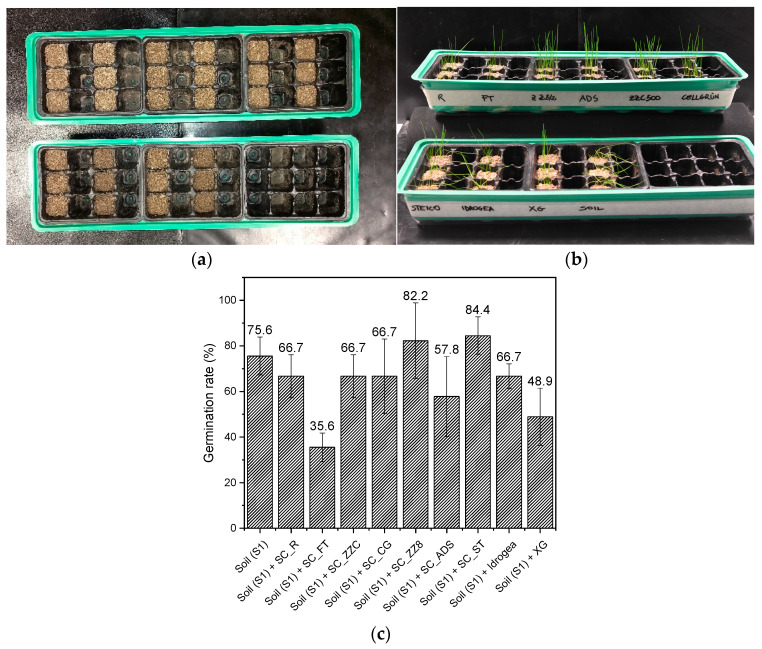
Representative images of (**a**) the grass seeds planted in the soil S1 treated with different SCs, (**b**) the grass growth after 18 days, and (**c**) the germination rate after 18 days.

In Figure 11a–c, the results of the grass cultivation experiments with the different soil conditioner compositions are displayed.

Figure 11b shows that grass fully develops in all types of soil samples after 18 days, demonstrating the non-harmful nature of the materials investigated. Additionally, Figure 11c indicates that the initial germination of seeds and the overall growth of sprouts are quite variable depending on the soil conditioner used. Indeed, the germination rate, defined as the ratio of the number of germinated seeds to the number of planted seeds, is higher for the soil treated with SC_ZZ8 and SC_ST with respect to the untreated soil. For the soil mixed with SC_R, SC_CG, SC_ZZC, and SC_ADS the germination rate is comparable to those of untreated soil and soil mixed with commercial products, while for the soil treated with SC_FT and with neat XG, it is lower. These results are quite coherent with those reported for water absorption and water retention tests. The most performant samples are those that show a good balance between good moisture absorption (Figure 6a), high WHC when mixed in the soil (Figure 7), and slow water release (Figure 6b and Figure 8). However, as already stated by Sorze et al. [68], these Xanthan-based composites are not designed to promote seed germination rate but are more suitable to support the growth of already hatched seedlings. Indeed, from Figure 11b, it is also possible to observe that the grass in pots with untreated soil is rather perished, while in the other pots, it is still thriving, despite the very low amount of daily water supplied. Therefore, this study confirms that the addition of cellulose fillers to Xanthan gum for the production of bio-based soil conditioners improves the water regulation capability of the soil, increasing the probability of plants survival under drought conditions. Due to the hydrophilic properties of their components, the prepared composites enhance the ability of soil to retain water, creating an environment that is more favorable for plant cultivation.

A possible limitation of these materials could result from the poor durability of the biopolymers in soil, which may affect their long-term efficiency. Although literature studies on the use of Xanthan in agricultural applications have shown that its performance in soil is still good even after 2 years [57], further investigations are needed to assess the applicability of these promising materials for long-term applications. Moreover, in view of a possible future upscaling, another potential limiting factor for these materials could be related to the current market price of this kind of biopolymer. However, the continuous increase in biopolymer production in recent years is expected to sensibly improve their economic feasibility. In particular, the material cost for 0.5% Xanthan gum for soil treatment has decreased from $250 to $28 in the last three decades [85]. Additionally, the Xanthan gum used in this work has food-grade quality with very high purity, which results in significantly higher production costs. For forestry and agricultural applications, edible grade purity is not necessary, and thus the price of this biopolymer is expected to be significantly lower if widely applied in these sectors.

## 4. Conclusions

In this work, soil conditioners based on Xanthan gum and different cellulose fillers were characterized to have a comprehensive evaluation of their influence on the water absorption, water retention, and water availability of soil. Light microscopy observations showed the homogeneous dispersion of these fillers within the Xanthan gum matrix, resulting in the formation of a reinforcing and stabilizing network. The water holding capacity of soil was increased from 34% (untreated soil) up to 69% (soil treated with 1.8 wt.% of SC_ST), with better performance in comparison to commercial products. Moreover, treated soil exhibited higher water retention with a water loss in drought conditions delayed by around two days with respect to untreated soil. Soil water retention curves, determined using the Hyprop evaporation and Dew Point Potentiometer methods, showed that the addition of SCs had beneficial effects on the following: (i) the available water for plants in the zone between field capacity and permanent wilting point; (ii) in cases of excess water, avoiding water stagnation; and (iii) in drought conditions, not increasing the pressure necessary to extract water from the soil with respect to the untreated soil. The results also confirmed the suitability of geotechnical investigations for the determination of the influence of SCs and showed a comparable influence of the SCs on water absorption, determined using two different methods on different soil samples. Finally, a case study application demonstrated the non-harmful nature of the SCs and highlighted the beneficial effect played by cellulose fillers on the water regulation properties of the soil.

## Figures and Tables

**Table 3:** List of prepared SCs samples.

Sample	Type of Filler
SC_R	Arbocel R
SC_FT	Arbocel FT 400
SC_ZZC	Arbocel ZZC 500
SC_CG	Cellugrün
SC_ZZ8	Arbocel ZZ 8-2 CA 1
SC_ADS	Arbocel Adsorb 2
SC_ST	STEICO flex 036
XG	-

**Table 4:** Values of yield stress for the prepared samples obtained through the Casson model (see Equation (1)).

Sample	Yield Stress [Pa]
SC_R	38.5 ± 1.6
SC_FT	56.9 ± 2.3
SC_ZZC	51.3 ± 2.5
SC_CG	44.2 ± 2.7
SC_ZZ8	37.7 ± 1.2
SC_ADS	35.5 ± 3.1
SC_ST	53.8 ± 1.8
XG	42.6 ± 2.2

**Table 5:** Water absorption of untreated and treated soil S2.

Specimen	Dosage of SC	w_A 24h_ (%)	w_A max_ (%) *
Soil (S2) (untreated)		87–89	87–89
Soil (S2) + SC_R	Low	91–99	Not measured
Soil (S2) + SC_R	High	154–162	166–176
Soil (S2) + SC_CG	High	140–150	174–201
Soil (S2) + SC_ZZC	High	131–139	140–175

* End of absorption: untreated soil 15 min, treated soil 2–4 days.

## Data Availability

Data are available on request by the corresponding author.
